# Markerless 3D hand tracking for analysis of pediatric eye-hand coordination^☆^

**DOI:** 10.1016/j.mex.2026.103965

**Published:** 2026-05-23

**Authors:** Anjali Rajkumar, Rajvardhan Gadde, Mahya Beheshti, Renat Sukhov, John-Ross Rizzo, Todd E. Hudson

**Affiliations:** a Department of Rehabilitation Medicine, NYU Langone Health, 244 E38th St., New York, NY 10016, USA; b Department of Neurology, NYU Langone Health, 222 E40th St., New York, NY 10016, USA; c Department of Biomedical Engineering, NYU Tandon School of Engineering, 433 First Avenue, New York, NY 10010, USA; d Department of Mechanical & Aerospace Engineering, NYU Tandon School of Engineering, 433 First Avenue, New York, NY 10010, USA

**Keywords:** Sensory-motor coordination, Pose estimation, Markerless limb tracking, Nine-Hole Peg Test, Pediatric rehabilitation

## Abstract

Precise quantification of eye-hand coordination (EHC) during pediatric dexterity tasks is limited by the lack of practical, high-resolution hand tracking methods suitable for children with brain injury or neurodegenerative disease. Traditional marker-based motion capture systems and instrumented gloves can interfere with natural grasp patterns and are often difficult to implement in clinical pediatric settings. We describe an adaptation of the Anipose markerless 3D pose estimation framework to enable synchronized three-dimensional hand kinematics and eye tracking during the Nine-Hole Peg Test (9HPT). The method integrates multi-camera video acquisition with task-specific neural network training optimized to detect fine finger movements across diverse pediatric hand sizes and grasp configurations. Camera placement and recording geometry were configured to reduce occlusion during peg manipulation and maintain multi-view visibility of hand landmarks. Model validation demonstrated low pixel error and stable three-dimensional reconstruction following confidence-based thresholding. The resulting workflow generates synchronized 2D and 3D visualizations, spatial coordinate outputs, reprojection-error metrics, and landmark confidence scores without requiring wearable sensors. This approach broadens the applicability of eye–hand coordination research within pediatric clinical populations and facilitates the development of more precise, quantitatively informed diagnostic assessments and targeted neurorehabilitation strategies for children with neurologic injury.

• Markerless multi-camera 3D reconstruction of pediatric hand kinematics during the 9HPT

• Integration of synchronized eye tracking and task-specific neural network training

• Output of validated 3D coordinates, confidence metrics, and visualization files suitable for clinical research

## Graphical abstract

**Figure:**
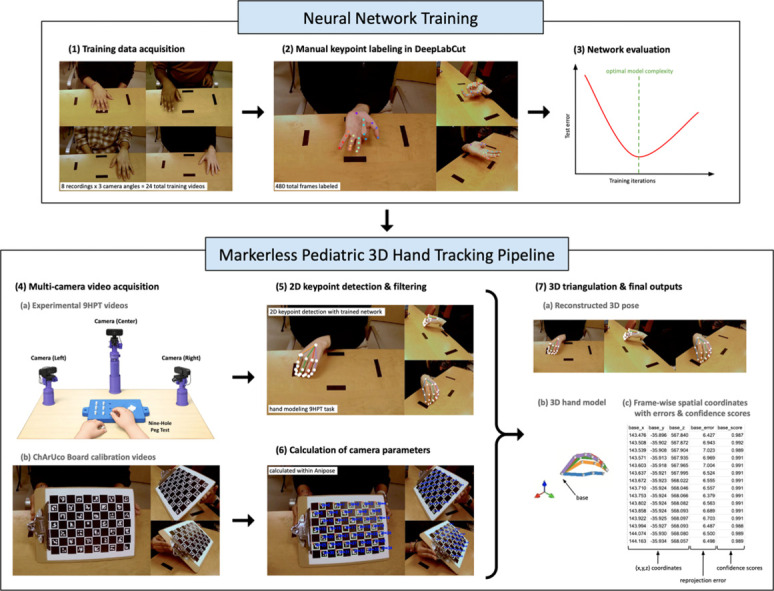

**Specifications table**

**Table:** 

**Subject area**	Neuroscience
**More specific subject area**	Eye-Hand Coordination
**Name of your method**	Markerless Pediatric 3D Hand Tracking
**Name and reference of original method**	*Anipose* Karashchuk, P., Rupp, K. L., Dickinson, E. S., Walling-Bell, S., Sanders, E., Azim, E., Brunton, B. W., & Tuthill, J. C. (2021). Anipose: A toolkit for robust markerless 3D pose estimation. Cell Reports, 36(13), 109,730. *https://doi.org/10.1016/j.celrep.2021.109730*
**Resource availability**	*3D models for top of camera mounts:*https://www.printables.com/model/6297-mini-tripod-for-dslr-and-digital-cameras/files *3D models for height adjustable body of camera mounts:*https://drive.google.com/drive/folders/1NgFM_NdQe6YT1Cn0jr_8OdwzuLNXP2-P?usp=sharing *Anipose (version 1.1.24)* *DeepLabCut GUI (version 2.3.11)*

## Background

Eye-hand coordination (EHC) is fundamental to goal-directed motor behavior and underlies everyday activities such as feeding, dressing, and object manipulation. Similar to adults, children with acquired brain injury (e.g., stroke or neurodegenerative disease) may exhibit impairments in eye–hand coordination due to disruptions across afferent sensory pathways, efferent motor systems, and integrative motor planning processes, thereby compromising the spatial and temporal coupling between gaze and hand movements [[1], [2], [3], [4]]. Such impairments are not fully captured by traditional clinical outcome measures and provide only limited information about the kinematic strategies used to perform tasks [5]. To better characterize functional limitations and recovery in pediatric populations, synchronized measurement of eye movements and detailed hand kinematics during standardized dexterity tasks is needed.

The Nine-Hole Peg Test (9HPT) is widely used to assess manual dexterity and upper extremity function [[6], [7], [8]]. Its primary outcome measure, task completion time, provides a global index of performance but does not capture how the task is executed. Completion time alone cannot describe grasp formation, hand posture, or the timing between gaze shifts and object manipulation. Integrating eye tracking with three-dimensional hand tracking during the 9HPT would enable precise quantification of visually guided motor behavior and provide a framework to distinguish maladaptive movement patterns from adaptive, compensatory strategies along a dyscoordination–coordination spectrum in children with brain injury or neurodegenerative disease.

While video-based eye tracking can be incorporated into structured motor assessments, high-resolution measurement of hand and finger motion has traditionally relied on marker-based motion capture systems or instrumented gloves [9]. Although these systems provide detailed kinematic data, they present practical challenges, particularly in pediatric populations. Gloves may not fit properly or may alter natural grasp patterns, while markers can detach, interfere with small object manipulation, and become a distraction for children. By contrast, a markerless approach preserves natural movement and minimizes participant burden, while still enabling detailed kinematic analysis.

The Anipose 3D pose estimation framework enables markerless reconstruction of movement from synchronized multi-camera video [10]. However, its original implementation did not specifically address the demands of tracking fine finger movements in small hands during pediatric dexterity tasks. Adapting this framework for the 9HPT requires optimization of camera placement, calibration procedures, and neural network training to enhance detection of small anatomical landmarks and mitigate occlusion during fine motor interactions such as peg manipulation.

In this study, we present an adaptation of the Anipose workflow for synchronized eye and hand tracking during the 9HPT in pediatric populations. The method integrates multi-camera video acquisition, two-dimensional keypoint detection using DeepLabCut, and three-dimensional reconstruction through triangulation. Camera positioning and training datasets were optimized to capture fine finger movements across a range of pediatric hand sizes, with network training focused on task-specific grasp and pinch configurations. By enabling synchronized, high-resolution measurement of gaze behavior and three-dimensional hand kinematics without wearable instrumentation, this approach advances the study of eye–hand coordination in pediatric brain injury and neurodegenerative disease and informs the development of more precise diagnostic and targeted rehabilitation strategies.

## Method details

**Fig. 1:**
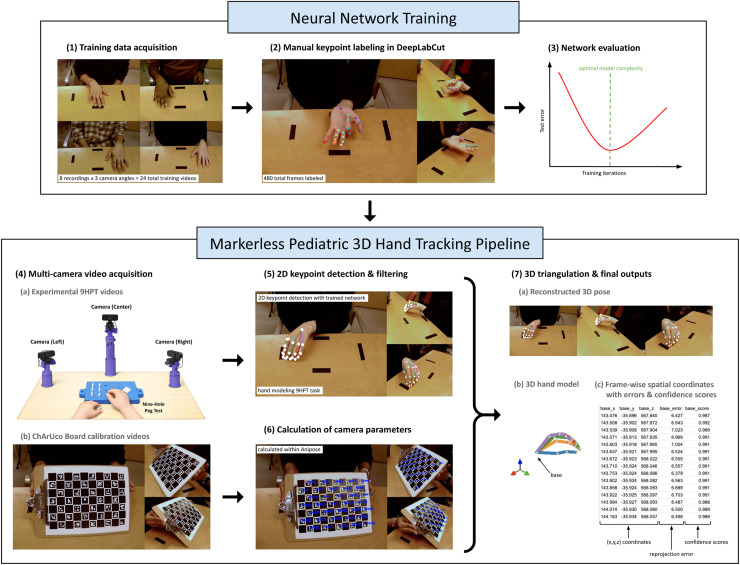
Markerless pediatric 3D hand tracking workflow.

To quantify hand kinematics during the 9HPT, we implemented a markerless multi-camera reconstruction workflow using Anipose. This workflow (see Fig. 1) combines multi-camera video acquisition, 2D keypoint detection using DeepLabCut, and 3D triangulation. Each stage of the procedure is described below.

All processing was performed using Anipose (version 1.1.24) within a Conda environment configured with Python 3.8.20, TensorFlow 2.8.0, and DeepLabCut 2.2.3 for integration within the Anipose pipeline. Neural network training and labeling through the DeepLabCut graphical user interface (GUI) were performed in a separate Conda environment using DeepLabCut 2.3.11 with Python 3.9.13 and TensorFlow 2.10.1 to ensure compatibility with the GUI requirements.

### Camera setup and calibration

**Fig. 2:**
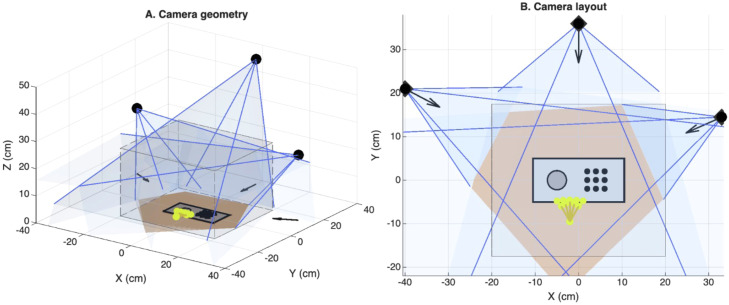
Multi-camera geometry for markerless reconstruction of hand kinematics during the Nine-Hole Peg Test. **(A)** Perspective view of the three-camera recording geometry used for markerless motion capture. A central camera positioned above the workspace provides a dorsal view of the hand, while two oblique cameras positioned laterally provide complementary views of finger motion. Camera frustums illustrate the fields of view of each camera, and the dashed box indicates the capture volume within which three-dimensional triangulation is performed. The task apparatus consists of a Nine-Hole Peg Test board with a peg basin and a 3 × 3 grid of peg holes. A schematic hand illustrates the typical reach trajectory from the peg basin to the pegboard during task performance. **(B)** Top-down view of the camera configuration showing the spatial arrangement of the three cameras relative to the task workspace. Arrows indicate the approximate viewing directions of the cameras. The shaded region represents the overlap of the camera fields of view corresponding to the region in which multi-view triangulation of hand landmarks is possible. This asymmetric camera arrangement increases baseline diversity and reduces occlusion during peg grasping and insertion, improving the robustness of three-dimensional hand landmark reconstruction. All coordinates are shown in centimeters.

Three USB cameras (640 × 480 pixel resolution, 20 fps) without fisheye lenses were positioned around the 9HPT workspace in a triangular configuration. The central camera was mounted 46 cm above the workspace and offset 36 cm posterior to the workspace center, with a downward angle of approximately 60° relative to the horizontal plane to capture a dorsal view of the hand (see Fig. 2). Two additional cameras were positioned laterally at a height of 24 cm and angled downward approximately 30°, providing oblique views of finger motion during peg grasping and insertion.

Relative to the workspace center, the left camera was positioned 40 cm to the left and 21 cm posterior, while the right camera was positioned 33 cm to the right and 14.5 cm posterior. This asymmetric arrangement provided complementary viewing angles while maintaining sufficient baseline separation for triangulation.

Cameras were mounted on stable supports to prevent movement during recording. The camera configuration was designed to ensure that all regions of the hand were visible in at least one view throughout peg grasping and placement. One camera was positioned centrally at an elevated height and angled downward to capture the dorsal surface of the hand. Two additional cameras were positioned diagonally to the left and right of the workspace and angled downward to capture lateral and frontal views. The capture volume was approximately 40 × 35 × 25 cm, encompassing the pegboard and the typical reach envelope of the participant’s hand during the task. Camera positions were specifically selected to optimize visibility of small finger movements during peg manipulation. This configuration also reduced occlusion of individual fingers during manipulation.

Camera calibration was performed using a 10 × 7 ChArUco board with a square side length of 25 mm and a marker side length of 18.75 mm. The board used the predefined OpenCV DICT_4 × 4_50 dictionary, comprising 50 unique 4 × 4 ArUco markers. A calibration video was recorded simultaneously from all three cameras while the board was moved slowly throughout the capture volume. The three video streams were synchronized using a custom script to trigger simultaneous recording initiation. The board was presented across the full field of view of each camera, including the edges and corners, and was briefly held at multiple depths and orientations. Anipose detected the ChArUco markers and calculated intrinsic and extrinsic camera parameters using bundle adjustment to minimize reprojection error [10]. Calibration was performed at the beginning of each recording session and repeated if any camera position changed.

#### Optimization of multi-camera geometry

Accurate three-dimensional reconstruction of fine hand movements requires camera placement that balances triangulation accuracy with robustness to occlusion. During the Nine-Hole Peg Test, finger landmarks frequently become obscured when digits overlap during grasp formation or when the hand rotates during peg insertion. To address these constraints, we implemented a three-camera configuration arranged in a triangular geometry around the workspace.

The central camera was positioned above and posterior to the pegboard and angled downward to capture a dorsal view of the hand and pegboard surface. This perspective provides stable visibility of the overall hand posture and the spatial relationship between the hand and task objects. Two additional cameras were placed diagonally to the left and right of the workspace and angled downward to capture oblique views of the hand. The lateral separation between the two oblique cameras was approximately 73 cm, providing sufficient baseline for stable triangulation across the workspace.

This multi-view configuration provides two key advantages for markerless reconstruction. First, the separation between cameras creates sufficient baseline distance for reliable triangulation of anatomical landmarks in three dimensions. Second, the overlapping fields of view ensure that each landmark remains visible in at least two cameras for many frames, even when temporary occlusion occurs in one view. This redundancy improves reconstruction stability and reduces the likelihood of tracking failure during rapid finger movements or complex hand postures.

Together, these camera placements provide robust multi-view coverage of the task workspace while maintaining a compact and easily deployable setup suitable for clinical environments.

#### Capture volume and task workspace

The capture volume was defined to encompass the pegboard and the typical reach envelope of the participant’s hand during the Nine-Hole Peg Test. In the present configuration, the effective capture region extended approximately 40 cm laterally, 35 cm in the anterior–posterior direction, and 25 cm vertically above the pegboard surface. This volume was selected to ensure that all phases of the task—including peg pickup, transport, and insertion—occurred within the overlapping fields of view of the three cameras. Maintaining the task within this shared viewing region allows hand landmarks to be observed from multiple perspectives throughout the movement sequence, supporting consistent three-dimensional reconstruction across the workspace.

#### Pediatric-specific design considerations

Tracking fine hand kinematics in pediatric populations introduces several challenges not typically encountered in adult motion capture. Children frequently exhibit greater variability in posture, hand orientation, and movement strategies during dexterity tasks, and may shift their body position while manipulating objects [11,12]. Smaller hand size and rapid changes in finger posture also increase the likelihood that individual landmarks will become occluded or briefly misidentified in video-based tracking.

**Fig. 3:**
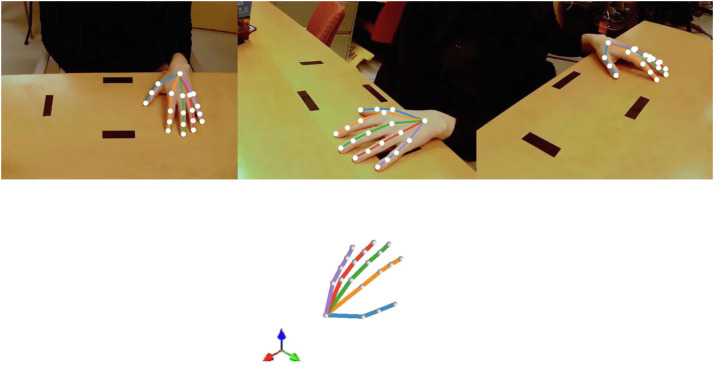
First frame of processed sample video with 2D detections and reconstructed 3D model shown.

To improve detection reliability under these conditions, several aspects of the system were optimized for pediatric participants. Training data for the keypoint detection model included hands of multiple sizes and movement patterns, with frames selected to capture diverse orientations and pinch configurations similar to those used during peg grasping. This task-specific dataset improves the model’s ability to identify anatomically consistent finger landmarks (Fig. 3) during fine manipulation.

An additional advantage of the present approach is its markerless design. Unlike marker-based motion capture systems or instrumented gloves, the method does not require children to wear sensors or specialized equipment. Avoiding wearable devices helps preserve natural grasp patterns and reduces participant burden, which is particularly important when working with children who may experience discomfort, distraction, or fatigue during testing. By minimizing interference with natural movement, the approach improves the feasibility and ecological validity of eye–hand coordination assessment in pediatric clinical populations.

### Training data acquisition

A separate dataset was collected to train the 2D keypoint detector. Four individuals were recorded moving their hands slowly within the capture volume to expose a range of positions, depths, and orientations. Movements included opening and closing the fist, isolated finger flexion and extension, forearm rotation, and thumb-index pinch movements similar to those used during the 9HPT. Two recordings were obtained of the left hand and two of the right hand for these movements. Four additional recordings were obtained of 9HPT task completion with the pegboard present, including two of the left hand and two of the right hand. All recordings were captured simultaneously from the three cameras, resulting in 24 training videos. Participants represented a range of skin tones to improve model generalizability.

### Neural network training

From these 24 training videos, 20 frames per video were extracted for manual labeling, resulting in 480 labeled frames. Frames were sampled to capture variability in hand posture, orientation, and depth across participants. Twenty anatomical landmarks were annotated per frame, including the base of the palm; the thumb (metacarpophalangeal joint, interphalangeal joint, and tip); and for each digit (index, middle, ring, and little fingers), the metacarpophalangeal, proximal interphalangeal, and distal interphalangeal joints, as well as the fingertip. Consistent anatomical definitions were applied across all frames and camera views.

The labeled dataset was divided into training and test sets within DeepLabCut, using a ResNet-50 network architecture, with 95% of manually labeled frames used to train the network and the remaining 5% used to test the model’s performance on unseen data. A 95/5 train-test split was selected to maximize training data while preserving an independent evaluation set. Model training was performed for up to 200,000 iterations. Training progress was displayed every 1000 iterations, and model snapshots were saved every 50,000 iterations. The four most recent snapshots were retained for evaluation. Model selection was based on minimizing test error. The first snapshot at 50,000 iterations demonstrated the lowest test error and was selected for subsequent processing.

### 2D keypoint detection and filtering

Experimental 9HPT task recordings were processed using the trained DeepLabCut network within Anipose to generate two-dimensional keypoint detections for each camera view. The resulting trajectories were refined using a series of complementary filtering steps [10]. A median filter was applied to reduce frame-to-frame jitter. A Viterbi-based temporal filter was used to promote trajectory continuity and reduce brief misidentifications. Finally, an autoencoder-based filter constrained predictions to anatomically plausible hand configurations. Filtered detections, as well as unfiltered ones if desired, were visually inspected by overlaying their keypoints onto the original video frames.

### 3D reconstruction and output generation

Three-dimensional positions were estimated by triangulating corresponding 2D detections across camera views using the calibrated camera parameters. For each frame, Anipose estimated the 3D position of each landmark (x, y, z) relative to the optical center of a chosen reference camera, which we defined as our centrally positioned camera. This camera was selected as the reference coordinate system because it maintained the most consistent view of the workspace and minimized perspective distortion relative to the pegboard. Triangulation was performed with optimization enabled (*optim* = true), enabling 3D filtering to refine reconstructed points. Anatomical consistency was enforced through a set of joint constraints linking adjacent landmarks along each digit (e.g., base–MCP–PIP–DIP–tip). Spatiotemporal smoothing and spatial regularization were applied using constraint scaling parameters (*scale_smooth* = 25; *scale_length* = 10) to promote trajectory continuity and preserve segment lengths [10]. The reconstructed 3D hand movements were then visualized in Anipose as a dynamic three-dimensional hand model over time [10]. The final output files included labeled 2D videos, 3D visualization videos, and frame-by-frame spatial coordinate files.

The accuracy of these reconstructed 3D coordinates was quantified using reprojection errors and confidence scores, both included in the spatial coordinate files. Reprojection error was calculated by Anipose as the Euclidean distance between the original 2D detections and their corresponding reprojected points, aggregated across camera views. This metric provides an internal measure of multi-camera consistency and overall reconstruction accuracy, reflecting the technical performance of the markerless multi-camera hand tracking workflow during the 9HPT. In addition to reprojection error, each anatomical landmark was assigned a confidence score representing the likelihood (ranging from 0 to 1) that the 2D tracking model correctly identified a landmark in a given frame, as produced by the underlying DeepLabCut network and carried forward into Anipose. Higher values indicate greater detection confidence. During 3D processing, Anipose uses these scores to guide filtering prior to triangulation. Specifically, low-confidence detections can be excluded or treated as missing and subsequently interpolated [10]. In our configuration, a score threshold of 0.5 was applied, such that detections with confidence values below 0.5 were treated as unreliable and removed during the 3D filtering stage.

Table 1 below summarizes the commands for each of the Anipose processing steps and their corresponding functions.

**Table 1:** Anipose commands and descriptions.

**Command**	**Function**
anipose analyze	videos are processed by DeepLabCut neural network to detect 2D keypoints from each camera view
anipose filter	2D keypoints are refined with the median filter, Viterbi-based temporal filter, and autoencoder-based filter
anipose label-2d-filter	filtered 2D detections overlaid on the original video frames
anipose label-2d	unfiltered 2D detections overlaid on the original video frames
anipose calibrate	computation of intrinsic and extrinsic camera parameters from each camera viewpoint to allow 3D reconstruction from 2D keypoints
anipose triangulate	filtered 2D keypoints are triangulated across cameras to estimate 3D pose in (x,y,z) coordinates with corresponding reprojection error metrics
anipose label-3d	reconstructed 3D pose are passed through an additional spatiotemporal filtering step to obtain refined 3D pose, then plotted to create a 3D hand model of hand movement over time
anipose label-combined	each group of videos obtained from “anipose label-2d” and the hand models obtained from “anipose label-3d” are linked to simultaneously show hand-tracking from each camera viewpoint

## Method validation

Method performance was evaluated at three stages of the workflow: [1] two-dimensional keypoint detection accuracy, [2] spatial accuracy of the three-dimensional reconstruction, and [3] representative outputs generated by the full workflow.

### Model selection and 2D keypoint detection performance

Neural network performance was evaluated using the training and test errors reported by DeepLabCut for each retained snapshot. As summarized in Table 2, training error progressively decreased as the number of iterations increased to 150,000; however, test error increased beyond 50,000 iterations, suggesting overfitting. The snapshot obtained at 50,000 iterations demonstrated the lowest test error after applying a confidence threshold (p-cutoff = 0.6) and was therefore selected for the final reconstruction pipeline using the *snapshotindex* parameter in Anipose.

**Table 2:** Network evaluation results.

**Training iterations**	**Train error** ***(px)***	**Test error** ***(px)***	**p-cutoff used**	**Train error with p-cutoff** ***(px)***	**Test error with p-cutoff** ***(px)***
50,000	3.1	7.35	0.6	3.08	5.08
100,000	2.99	7.97	0.6	2.99	5.57
150,000	2.55	7.35	0.6	2.55	5.26
200,000	3.0	7.44	0.6	3.0	5.57

At 50,000 iterations, the training error was 3.1 pixels and the test error was 7.35 pixels prior to applying the confidence threshold. After applying the p-cutoff of 0.6, the training error decreased slightly to 3.08 pixels and the test error decreased to 5.08 pixels. These values represent the mean Euclidean pixel distance between predicted and manually labeled keypoint positions in the respective datasets. The reduction in test error following thresholding indicates that excluding low-confidence detections improved overall prediction reliability.

To provide a physical interpretation of this error magnitude, pixel-level deviations were converted to spatial units based on the camera field of view and working distance. Under the present recording geometry, the imaging region captured by the central camera corresponds to approximately 0.35–0.45 mm per pixel at the workspace surface. Consequently, the network’s 2D keypoint detection error of 5 pixels corresponds to a spatial uncertainty of approximately 1.75–2.25 mm within the capture volume, which is sufficient to resolve finger joint motion and pinch formation during peg manipulation.

### Validation of 3D reconstruction accuracy

To evaluate the spatial accuracy of the markerless reconstruction pipeline, reprojection error was computed following triangulation of the filtered two-dimensional detections. As described previously, reprojection error quantifies the difference between observed two-dimensional keypoints and the projections of their reconstructed three-dimensional positions back into each camera view.

A representative recording was processed using the selected model to assess reconstruction performance across a full sequence of movements. The video included a variety of hand movements including opening and closing the fist, isolated finger flexion and extension, forearm rotation, and thumb-index pinch movements similar to those used during the 9HPT. Reprojection error was computed across all anatomical landmarks and frames in this representative recording. The mean reprojection error was 6.54 pixels, corresponding to approximately 2.28–2.94 mm of spatial uncertainty within the capture volume. This value is comparable to the two-dimensional keypoint detection error (5.08 pixels, approximately 1.8–2.3 mm), indicating that uncertainty remains of similar magnitude after triangulation and is not substantially amplified during the three-dimensional reconstruction process.

This level of spatial precision is sufficient to resolve the reach trajectories and grasp configurations associated with the Nine-Hole Peg Test, where hand movements typically span several centimeters and individual peg manipulations occur over distances of approximately 1–2 cm. Additionally, because multiple cameras observe each landmark from different viewing angles, transient occlusion in a single camera view does not often result in reconstruction failure.

### Representative pipeline output

The same representative recording used for the reconstruction accuracy analysis was used to illustrate the outputs generated by the full reconstruction workflow. Table 3 reports the reconstructed 3D spatial coordinates, reprojection errors, and confidence scores for each anatomical landmark in the first frame of the recording. Fig. 1 shows the corresponding output from the final processing stage, including filtered 2D detections overlaid on the original videos from each camera view and the reconstructed 3D hand model. Together, these results demonstrate that the workflow provides stable millimeter-scale reconstruction accuracy suitable for quantifying fine hand movements during dexterity tasks.

**Table 3:** Spatial coordinates with reprojection errors for the first frame of sample video.

**Landmark**	***x* (px)**	***y* (px)**	***z* (px)**	**Error (px)**	**Score**
**Base**	143.476	−35.896	567.840	6.427	0.987
**MCP 1**	88.476	−12.833	556.660	2.645	0.983
**PIP 1**	72.917	7.013	547.358	3.020	0.999
**Tip 1**	57.918	29.519	538.106	0.851	1.000
**MCP 2**	112.640	9.740	520.738	6.640	0.993
**PIP 2**	107.035	46.390	507.485	3.378	0.999
**DIP 2**	104.427	68.161	503.959	2.018	1.000
**Tip 2**	101.318	87.131	501.556	2.447	0.999
**MCP 3**	138.093	14.388	522.687	6.359	0.997
**PIP 3**	135.494	50.709	507.604	1.265	0.994
**DIP 3**	133.651	74.698	501.512	2.883	0.998
**Tip 3**	133.511	94.372	497.266	3.093	0.993
**MCP 4**	155.945	13.778	531.061	4.906	0.996
**PIP 4**	157.884	46.333	517.028	2.119	0.995
**DIP 4**	157.978	68.593	510.298	2.431	0.999
**Tip 4**	157.300	87.700	504.789	4.097	0.999
**MCP 5**	168.311	11.543	543.014	5.027	0.989
**PIP 5**	175.454	39.006	533.825	3.158	0.994
**DIP 5**	178.583	55.911	527.309	3.450	0.998
**Tip 5**	182.188	72.297	520.191	3.128	0.999

In summary, this work presents a markerless workflow for reconstructing three-dimensional hand kinematics during the Nine-Hole Peg Test. By combining task-specific neural network training, optimized multi-camera geometry, and the Anipose pipeline, the method achieves millimeter-scale spatial resolution without requiring wearable sensors or gloves, enhancing feasibility and ecological validity in pediatric populations while preserving natural grasp behavior.

Future work will integrate this markerless hand tracking workflow with synchronized eye-tracking measurements to enable simultaneous analysis of gaze behavior and fine motor kinematics during dexterity tasks. These combined measurements will allow high-resolution characterization of eye–hand coordination and provide a framework to quantify performance along a dyscoordination–coordination spectrum, supporting the development of more precise diagnostic assessments and targeted, mechanism-informed rehabilitation strategies for children with neurologic injury or neurodegenerative disease.

## Limitations

Accurate three-dimensional reconstruction is dependent on stable camera positioning; small shifts in camera placement may affect calibration parameters and require recalibration prior to analysis. In addition, optimal performance of the DeepLabCut-based keypoint detector depends on task-specific training, and retraining may be required when there are significant changes in subject appearance or recording conditions (e.g., lighting or background), when new or distinct hand postures are introduced, or when camera angles or the experimental setup are modified, in order to maintain reconstruction accuracy.

## CRediT author statement

**Anjali Rajkumar:** Methodology, Software, Investigation, Formal analysis, Writing - Original Draft, Visualization. **Rajvardhan Gadde:** Methodology, Software, Investigation, Writing - Review & Editing. **Mahya Beheshti:** Writing - Review & Editing, Supervision. **Renat Sukhov:** Conceptualization, Supervision. **John-Ross Rizzo:** Conceptualization, Writing - Review & Editing, Supervision. Todd E. **Hudson:** Conceptualization, Writing - Review & Editing, Visualization, Project administration.

## Ethics statements

Not applicable.

## Declaration of competing interest

The authors declare that they have no known competing financial interests or personal relationships that could have appeared to influence the work reported in this paper.

## Data Availability

Data will be made available on request.
